# Screening for deafness-associated mitochondrial 12S rRNA mutations by using a multiplex allele-specific PCR method

**DOI:** 10.1042/BSR20200778

**Published:** 2020-05-28

**Authors:** Yu Ding, Jianyong Lang, Junkun Zhang, Jianfeng Xu, Xiaojiang Lin, Xiangyu Lou, Hui Zheng, Lei Huai

**Affiliations:** 1 Central laboratory, Hangzhou First People’s Hospital, Zhejiang University School of Medicine, Hangzhou, China; 2 Department of Otolaryngology, Fuyang Second People’s Hospital, Hangzhou, China; 3 Department of Otolaryngology, Chun’an Traditional Chinese Hospital, Hangzhou, China; 4 Department of Otolaryngology, Jiande Second People’s Hospital, Hangzhou, China; 5 Department of Otolaryngology, Kaihua People’s Hospital, Quzhou, China; 6 Department of Otolaryngology, Hangzhou First People’s Hospital, Zhejiang University School of Medicine, Hangzhou, China

**Keywords:** Chinese pedigrees, deafness, MAS-PCR, mitochondrial 12S rRNA mutations

## Abstract

Mitochondrial 12S rRNA A1555G and C1494T mutations are the major contributors to hearing loss. As patients with these mutations are sensitive to aminoglycosides, mutational screening for 12S rRNA is therefore recommended before the use of aminoglycosides. Most recently, we developed a novel multiplex allele-specific PCR (MAS-PCR) that can be used for detecting A1555G and C1494T mutations. In the present study, we employed this MAS-PCR to screen the 12S rRNA mutations in 500 deaf patients and 300 controls from 5 community hospitals. After PCR and electrophoresis, two patients with A1555G and one patient with C1494T were identified, this was consistent with Sanger sequence results. We further traced the origin of three Chinese pedigrees. Clinical evaluation revealed variable phenotypes of hearing loss including severity, age at onset and audiometric configuration in these patients. Sequence analysis of the mitochondrial genomes from matrilineal relatives suggested the presence of three evolutionarily conserved mutations: tRNA^Cys^ T5802C, tRNA^Lys^ A8343G and tRNA^Thr^ G15930A, which may result the failure in tRNAs metabolism and lead to mitochondrial dysfunction that was responsible for deafness. However, the lack of any functional variants in *GJB2, GJB3, GJB6* and *TRMU* suggested that nuclear genes may not play active roles in deafness expression. Hence, aminoglycosides and mitochondrial genetic background may contribute to the clinical expression of A1555G/C1494T-induced deafness. Our data indicated that the MAS-PCR was a fast, convenience method for screening the 12S rRNA mutations, which was useful for early detection and prevention of mitochondrial deafness.

## Introduction

Hearing loss is a very common human health problem, affecting approximately 360 million people worldwide and more than 27 million individuals in China [1]. Most hearing loss is non-syndromic, but deafness can also be associated with other abnormalities, which was called syndromic hearing loss. In fact, hearing loss can be caused by environmental factors or genetic factors, of which mitochondrial DNA (mtDNA) mutation plays a critical role in aminoglycoside-induced and non-syndromic hearing loss (AINSHL) [2]. In particular, mitochondrial 12S rRNA gene is the hot spot for pathogenic mutations associated with deafness [3]. Among them, the A1555G and C1494T mutations have been implicated to be linked with AINSHL in many families worldwide [4,5]. Notice that the A1555G/C1494T mutation creates an extremely conserved 1494-1555G-C or 1494-1555A-U base-pairing at the A-site of mitochondrial 12S rRNA where the codon and anticodon recognition occurs [6]. This transition makes the human mitochondrial ribosome more bacteria-like, and consequently alters binding sites for aminoglycoside antibiotics (AmAn) [7]. Thus, screening the two primary mutations of deafness in general population is important for genetic counseling and disease prevention [8]. To date, several molecular methods have been designed for detecting the deafness-associated gene mutations, such as denaturing high-performance liquid chromatography (DHPLC) [9], SNaPshot mini-sequencing technology [10], amplification refractory mutation system PCR (ARMS-PCR) [11] and PCR-Sanger sequencing. However, these methods are complex, cost-ineffectiveness and hindered by the requirement of high-end instruments, thus cannot be widely used in early detection and clinical diagnosis for hearing loss.

With this regard, we recently developed a novel multiplex allele-specific PCR (MAS-PCR) for molecular detecting the deafness-associated 12S rRNA mutations [12]. We first designed 4 primers that specifically binding to human 12S rRNA gene, after PCR amplification and electrophoresis, patients carrying the A1555G mutation resulted in two specific bands: 736-bp and 226-bp, while subjects with the C1494T mutation created two bands: 736-bp and 488-bp, whereas patients without these primary mutations can amplify only one band: 736-bp. To further assess its accuracy, we applied this method by examining the presence of mitochondrial A1555G or C1494T mutation in 200 patients with hearing impairment and 120 controls, as expected, the data were well consistent with the results of DNA sequencing (*Kappa* = 1.000, *P*<0.01) [13]. Therefore, this MAS-PCR was a simple, reliable and useful method that can be used to detect the deafness-related A1555G or C1494T mutation.

In the present study, with the purpose of prevention the incidence of mitochondrial deafness and providing valuable information for molecular diagnosis of hearing loss, we employed our MAS-PCR to screen the presence of A1555G or C1494T mutation in 500 deaf patients and 300 controls in 5 community hospitals from Zhejiang Province, P.R. China. As a result, 2 patients with A1555G and 1 patient carrying C1494T mutations were identified, which was consistent with the results of PCR-Sanger sequencing. Moreover, we performed the clinical and molecular analysis of 3 Chinese pedigrees with mitochondrial 12S rRNA mutations. Sequence analysis of the entire mitochondrial genomes from the matrilineal relatives suggested the presence of tRNA^Cys^ T5802C, tRNA^Lys^ A8343G and tRNA^Thr^ G15930A mutations.

## Materials and methods

### Subjects

From January 2015 to January 2018, a total of 500 deaf patients (263 males and 237 females, aged from 21 to 65 years, with an average of 42 years), together with 300 controls (169 males and 131 females, aged from 19 to 55 years, with an average of 39 years) were recruited from 5 community hospitals from Zhejiang Province of P.R. China: Hangzhou First People’s Hospital; Fuyang Second People’s Hospital; Chun’an Traditional Chinese Hospital; Jiande Second People’s Hospital and Kaihua People’s Hospital. The present study was conducted in accordance with the Declaration of Helsinki. Written informed consent was acquired before the study from all participants or their parents, and the study protocols were ratified by the Ethics Committee of Hangzhou First People’s Hospital, Zhejiang University School of Medicine.

### MAS-PCR

The genomic DNA from each deaf patient together with 300 controls was extracted from venous blood by a TIANamp Blood DNA Kit (TianGen Biotech Co. Ltd., Beijing, China), the DNA’s concentration was measured and stored at −30°C until further use. Four primers used to amplify wild-type version of 12S rRNA; 12S rRNA with A1555G mutation and 12S rRNA with C1494T mutation were designed by Primer Premier 5.0 software. The sequences of these primers were as follows: 5′-AAGTGGCTTTAACATATCTG-3′; 5′-TTGAAGTATACTTGAGCAGA-3′; 5′-ACGCATTTATATAGAGCAGG-3′ and 5′-TCAATTTCTATCGCCTATAC-3′ [12].

The PCR mixture (20 μl) contained 10x Buffer (with Mg^2+^), Ex Taq DNA Polymerase 0.5 units, dNTP 175 μmol/l, 10 μmol/l each primer and 20 ng/μl DNA temple. The PCR was performed by using the following conditions: 94°C for 5 min; 5 amplification cycles of 94°C for 40 s, 53°C for 40 s, and 72°C for 40 s, 1°C reduction of annealing temperature at the end of each cycle; then 25 cycles of 94°C for 40 s, 48°C for 40 s, 72°C for extension 40 s, and a final extension cycle at 72°C for 7 min. After that, PCR product (5 μl) was analyzed by using 1.5% agarose gel electrophoresis at 130 V for 30 min.

### Genotyping analysis of 12S rRNA mutations by PCR-Sanger sequencing

We further performed PCR and direct sequence analysis to confirm the presence of mitochondrial A1555G or C1494T mutation. The primers’ information for genetic amplification of mitochondrial 12S rRNA gene was mentioned in elsewhere [14]. After PCR, the product was purified and analyzed by Sanger sequencing in an ABI 3700 automated DNA sequencer. The data were compared with the revised Cambridge References sequence (rCRS) to detect the mutations (GenBank accession number: NC_012920.1) [15].

### Characterization of three Chinese families with AINSHL

**Figure 1:**
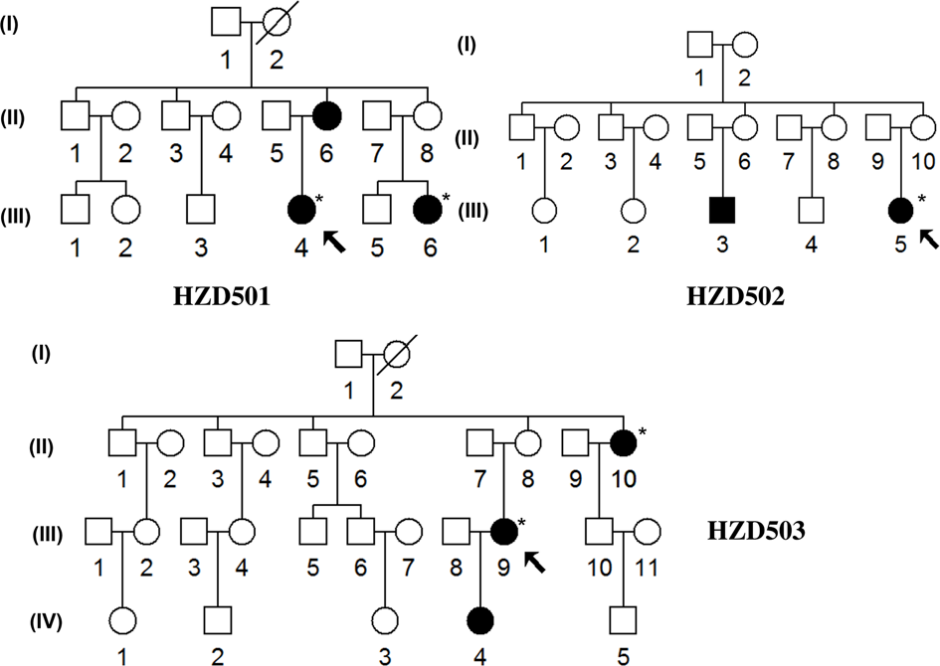
Three Chinese families with AINSHL The affected members were indicated by filled symbols, arrows denoted the probands, asterisks suggested the individuals who had a history of using AmAn.

In this case–control study for genetic screening of deafness-associated 12S rRNA mutations, three Han Chinese pedigrees with AINSHL (Family ID: HZD501; HZD502 and HZD503) were ascertained in the Department of Otolaryngology, Hangzhou First People’s Hospital (Figure 1). A comprehensive history of each family member was obtained using a questionnaire including the age at onset of hearing loss, the level of hearing impairment, the history of using AmAn, noise exposure and other clinical disorders.

Moreover, the pure-tone audiometry (PTA) with air and bone conduction was tested according to standard protocols in a sound-controlled room at frequencies ranging from 250 to 8000 Hz. The severity of hearing impairment was classified into five grades: normal <26 decibels (dB); mild: 26–40 dB; moderate: 41–70 dB; severe: 71–90 dB; and profound >90 dB. Notice that the penetrance of hearing loss was calculated by dividing the affected number of matrilineal relatives by the total number of matrilineal relatives.

### Screening for the whole mitochondrial mutations

Since the maternally transmission of hearing impairment in these pedigrees (HZD501, HZD502 and HZD503), which indicated that mtDNA dysfunctions caused by mtDNA mutations/variants may be involved in the pathogenesis of hearing loss. For this purpose, we performed PCR amplification of the entire mitochondrial genomes of the matrilineal relatives (HZD501: II-6, III-4 and III-6; HZD502: III-3 and III-5; HZD503: II-10, III-9 and IV-4), as well as 300 control subjects, according to the protocol described previously [16]. The PCR products were purified and analyzed by direct sequencing in an ABI 3700 automated DNA sequencer. The sequence data were compared with the rCRS (GenBank accession number: NC_012920.1) to detect the mutations or variants [15].

### Phylogenetic conservation analysis

A total of 17 vertebrate mtDNA sequences from NCBI databases (https://www.ncbi.nlm.nih.gov/) were used for the phylogenetic analysis. We further calculated the conservation index (CI) of each mtDNA variant, the CI was defined as the percentage of the assessed species having the human wild-type nucleotide at the specified position. The CI ≥ 75% was regarded as having functional potential [17].

### Analysis of mitochondrial haplogroups

The phylogenetic trees were used to determine the haplogroups, including the mtDB (http://www.genpat.uu.se/mtDB ) and the updated East Asian mtDNA phylogeny [18].

### Bioinformatics analysis

To test whether mitochondrial tRNA (mt-tRNA) mutations affected the tRNA function, we utilized RNA Fold Webserver (http://rna.tbi.univie.ac.at/cgi-bin/RNAfold.cgi) to predict the secondary structure of tRNA^Cys^, tRNA^Lys^ and tRNA^Thr^ with and without T5802C, A8343G and G15930A mutation, respectively [19]. In addition to minimum free energy (MFE) folding, equilibrium base-pairing probabilities were calculated via John McCaskill’s partition function (PF) algorithm [20].

### Prediction the pathogenicity of mt-tRNA mutations

We used the updated pathogenicity scoring system to assess the pathogenic status of three mt-tRNA mutations (tRNA^Cys^ T5802C, tRNA^Lys^ A8343G and tRNA^Thr^ G15930A) identified in the present study [21]. Based on that standard, a mt-tRNA variant was regarded as ‘neutral polymorphism’ if its score was ≤ 6 points, if the score ranked between 7 and 10 points, it belonged to ‘possible pathogenic’, whereas the score was ≥11 points, the variant was classified as ‘definitely pathogenic’.

### Mutational analysis of deafness-associated nuclear genes

To analysis the contributions of nuclear genes (*GJB2, GJB3, GJB6* and *TRMU*) in the phenotypic manifestation of A1555G or C1494T-induced deafness, we performed a mutational analysis of these common nuclear genes from the matrilineal relatives (HZD501: II-6, III-4 and III-6; HZD502: III-3 and III-5; HZD503: II-10, III-9 and IV-4) by using the methods as described previously [22]. After PCR amplification and direct Sanger sequence, the data were compared with the wild-type versions of *GJB2, GJB3, GJB6* and *TRMU* sequences (GenBank accession numbers: M86849, AF052692, NG_008323 and AF_448221, respectively) to detect the mutations or variants.

### Statistical analysis

The SPSS 21.0 software (SPSS Inc., Chicago, IL, U.S.A.) was used to analysis the data, the Fisher’s exact test and the Kappa statistics were performed, a *P* value of 0.05 or less was considered statistically significant. The following values for strength of agreement for Kappa were considered: poor (<0.21); fair (0.21–0.40); moderate (0.41–0.60); good (0.61–0.80) and very good (0.81–1.00).

## Results

### Screening for mitochondrial A1555G and C1494T mutations using MAS-PCR

**Figure 2:**
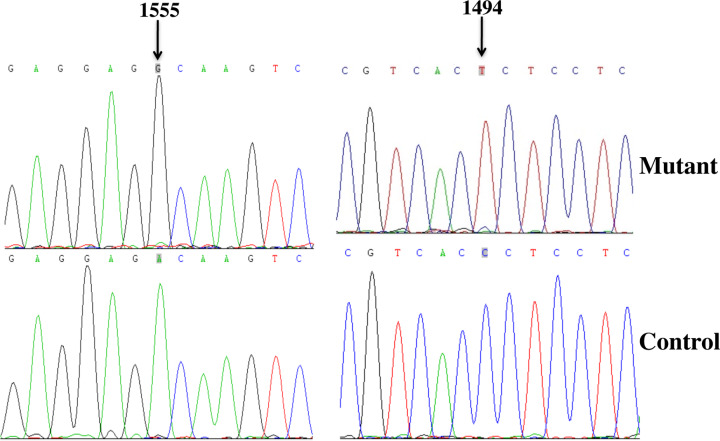
Identification of mitochondrial A1555G and C1494T mutations by using direct sequencing

We carried out a screening for deafness-associated 12S rRNA mutations in 500 deaf patients and 300 controls by using the MAS-PCR that had been successfully established in our laboratory [12]. Consequently, two patients with A1555G (0.4%) and one patient with C1494T (0.2%) mutations were identified based on the electrophoresis results (data not shown). The PCR results were completely concordant with the direct sequencing data of the three individuals (Table 1 and Figure 2), and had a high sensitivity and specificity. However, these primary mutations were not detected in 300 healthy subjects (*Kappa* = 1.000, *P*<0.01).

**Table 1:** Comparison of MAS-PCR and direct Sanger sequencing

MAS-PCR	Sanger sequencing			Total
	Wild type (12S rRNA) (*n*)	A1555G mutation (*n*)	C1494T mutation (*n*)	
Wild-type (12S rRNA)	497	0	0	497
A1555G mutation	0	2	0	2
C1494T mutation	0	0	1	1
Total	497	2	1	500

Kappa = 1.000, *P*<0.01

### Clinical characterization of three Han Chinese families with hearing impairment

**Figure 3:**
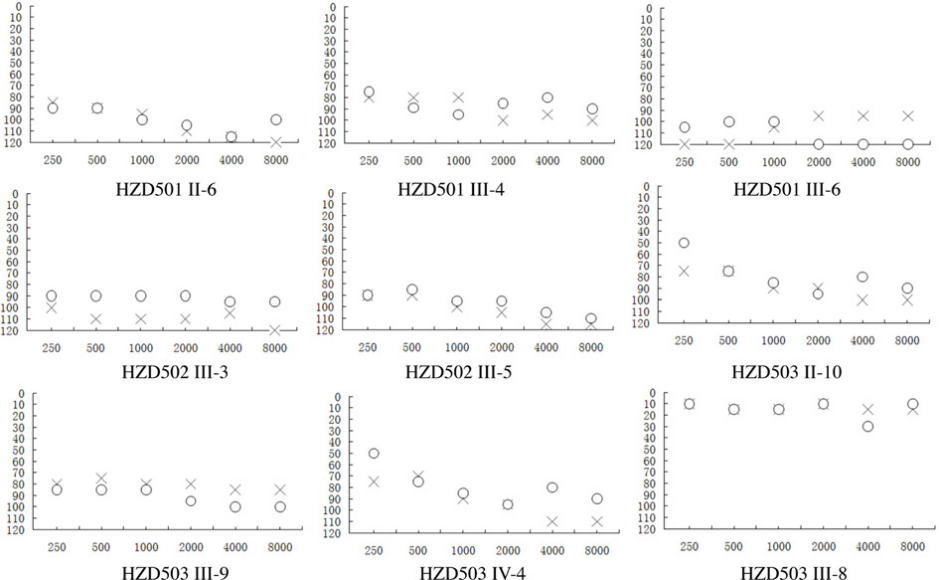
Audiograms of several matrilineal relatives in three Chinese pedigrees (HZD501, HZD502 and HZD503), X: left ear; O: right ear

In the family HZD501, the proband (III-4) was a 41-year-old woman who came to Hangzhou First People’s Hospital for treatment of deafness. She was received gentamycin for high fever when she was 16. Unfortunately, she began to suffer bilateral hearing loss 10 days after the drug administration. As shown in Figure 3 and Table 2, audiological evaluation showed that she had severe hearing loss (86 dB at left ear and 85 dB at right ear). A comprehensive family history revealed that the proband's mother (II-6) and sister (III-6) were also deaf patients. The family member (III-6) was treated with neomycin when she was 21, whereas other members in HZD501 had normal hearing.

**Table 2:** Summary of clinical data for several members in three pedigrees with hearing loss

Subjects	Gender	Use of AmAn	Age at test (year)	Age at onset (year)	PTA (Left ear) (dB)	PTA (Right ear) (dB)	Level of hearing loss
**HZD501 II-6**	Female	No	66	60	101	100	Profound
**HZD501 III-4**	Female	Yes	41	16	86	85	Severe
**HZD501 III-6**	Female	Yes	35	21	102	110	Profound
**HZD502 III-3**	Male	No	21	19	108	91	Profound
**HZD502 III-5**	Female	Yes	25	10	98	96	Profound
**HZD503 II-10**	Female	Yes	77	51	88	78	Severe
**HZD503 III-9**	Female	Yes	41	34	80	91	Profound
**HZD503 IV-4**	Female	No	16	8	90	78	Severe
**HZD503 III-8**	Male	No	41	/	13	16	Normal

In the pedigree HZD502, the proband (III-5) was a 25-year-old woman who lived in Hangzhou area from Zhejiang Province. She went to Hangzhou First People’s Hospital for treatment of deafness. As can be seen in Figure 3 and Table 2, she was diagnosed as profound hearing loss (98 dB at left ear and 96 dB at right ear). After the genetic counseling, we found that she was received the gentamycin for treatment of fever when she was 10. Notably, another family member (III-3) was also a deafness carrier (108 dB at left ear and 91 dB at right ear).

The proband (III-9) of the family HZD503 lived in Hangzhou City from Zhejiang Province, as shown in Figure 3, she was diagnosed as profound hearing loss. She was administrated with gentamycin for treatment of fever when she was 34, 1 week later; she developed profound hearing loss (80 dB at left ear and 91 dB at right ear). Moreover, the family members (II-10 and IV-5) were deafness carriers, while the member (II-10) had the history of using AmAn. The clinical data for each deaf patient from these families were listed in Table 2.

Interestingly, these Chinese families exhibited different penetrances of hearing loss. Notice that if the AmAn was included, the penetrance of hearing loss in HZD501, HZD502 and HZD503 was 42.8%, 22.2% and 37.5%, respectively. However, if the AmAn was excluded, the penetrance of hearing loss in HZD501, HZD502 and HZD503 was 14.3%, 11.1% and 12.5%, respectively, suggesting that AmAn was an important risk factor for hearing loss.

### mtDNA sequence analysis

**Figure 4:**
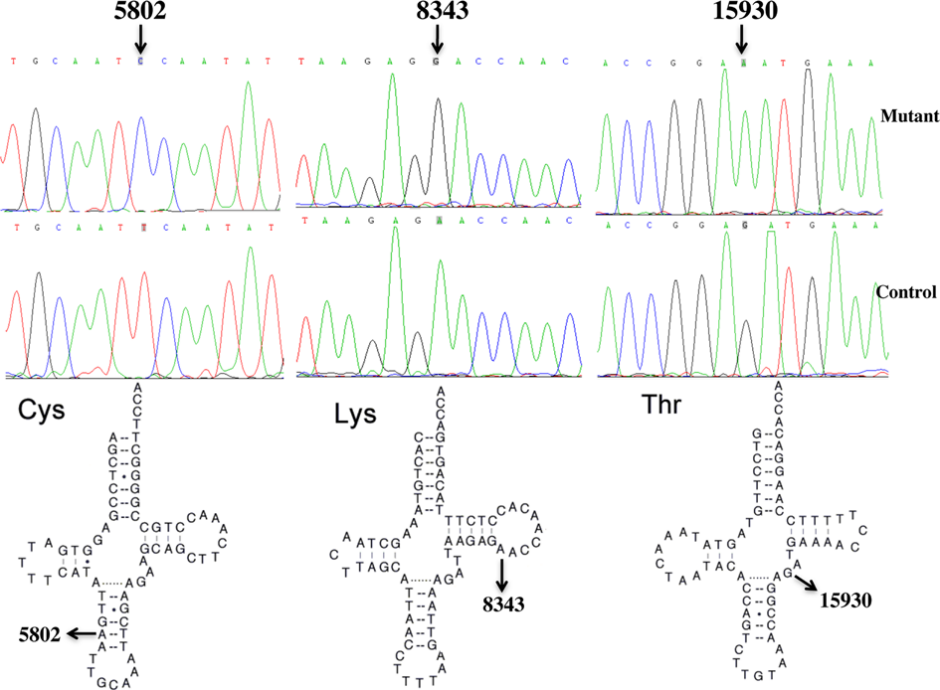
Identification of tRNA^Cys^ T5802C, tRNA^Lys^ A8343G and tRNA^Thr^ G15930A mutations by Sanger sequencing The secondary structures of tRNA^Cys^, tRNA^Lys^ and tRNA^Thr^ were derived from Mitomap database (www.mitomap.org). Arrows indicated the locations of T5802C, A8343G and G15930A mutations.

Since these families were maternally transmitted (Figure 1), which indicated that mtDNA mutations or variants played active roles in the phenotypic manifestation of hearing impairment. For this purpose, we performed PCR amplification of the complete mitochondrial genomes of the matrilineal relatives (HZD501: II-6, III-4 and III-6; HZD502: III-3 and III-5; HZD503: II-10, III-9 and IV-4) according to the methods as described in elsewhere [16]. Consequently, the mutations of mitochondrial genomes sequences were screened and detected (Table 3), these subjects from HZD501, HZD502 and HZD503 exhibited distinct sets of mtDNA single-nucleotide polymorphisms (mtSNPs) that belonged to mtDNA haplogroup B4b1c, D4b2b and K1a, respectively [18]. Among these SNPs, there were 23 variants in D-loop gene, 7 variants or mutations in 12S rRNA and 3 variants in 16S rRNA, 3 mutations in mt-tRNA genes, as well as the common CO2/tRNA^Lys^ intergenic 9-bp deletion corresponding with mtDNA at positions 8271–8279. Besides these mutations/variants, others SNPs mainly occurred at the oxidative phosphorylation (OXPHOS)-encoding genes. Moreover, 10 missense mutations were identified, including *ND1* G3391C (Gly to Ser), *ND2* G4491A (Val to Ile) and C5178A (Leu to Met), *CO2* T7785C (Ile to Thr), *A6* C8414T (Leu to Phe), A8701G (Thr to Ala) and A8860G (Thr to Ala), *ND3* A10398G (Thr to Ala), *CytB* C14766T (Thr to Ile) and A15326G (Thr to Ala). Most of these SNPs were well-known mutational hot spots and none of these variants could be classified as ‘novel’ [23]. These variants in rRNAs, tRNAs or polypeptides were further evaluated by phylogenetic analysis of sequences from other organisms including the mouse [24], bovine [25] and *Xenopus laevis* [26]. Our results showed that the A1555G, C1494T, tRNA^Cys^ T5802C, tRNA^Lys^ A8343G and tRNA^Thr^ G15930A mutations were very conserved among various species (Figure 4), whereas other variants showed no evolutionary conservation. In addition, genetic screening of these mutations indicated that the A1555G, C1494T, T5802C, A8343G and G15930A mutations were not detected in 300 controls (*P*<0.05 for all), suggesting that they may have functional potential.

**Table 3:** mtDNA sequence variants in three Chinese families with hearing impairment

Gene	Position	Alternation	Conservation (H/B/M/X)^a^	rCRS^b^	HZD501	HZD502	HZD503	Previously reported^c^
**D-loop**	73	A to G		A	G	G	G	Yes
	150	C to T		C	T		T	Yes
	215	G to A		G	A			Yes
	249	A to G		A		G		Yes
	263	A to G		A	G		G	Yes
	310	T to C		T	C	C	C	Yes
	489	T to C		T	C	C		Yes
	499	G to A		G			A	Yes
	523	Del A		A	Del A			Yes
	524	Del C		C		Del C		Yes
	573	C to T		C	C		C	Yes
	16051	A to G		A	G			Yes
	16093	T to C		T			C	Yes
	16129	G to A		G			A	Yes
	16136	T to C		T	C			Yes
	16154	T to C		T		C		Yes
	16172	T to C		T			C	Yes
	16189	T to C		T	C	C	C	Yes
	16223	C to T		C	T			Yes
	16234	C to T		C		T		Yes
	16266	C to T		C			T	Yes
	16362	T to C		T		C		Yes
	16519	T to C		T	C	C		Yes
**12S rRNA**	750	A to G	A/G/G/-	A	G	G	G	Yes
	827	A to G		A	G			Yes
	1041	A to G		A			G	Yes
	1382	A to G	A/A/A/G	A		G		Yes
	1438	A to G	A/A/A/G	A	G	G	G	Yes
	1494	C to T	C/C/C/C	C	T			Yes
	1555	A to G	A/A/A/A	A		G	G	Yes
**16S rRNA**	2706	A to G	A/G/A/A	A	G	G	G	Yes
	3010	G to A	G/G/A/A	G		A		Yes
	3107	Del N		N	Del N	Del N	Del N	Yes
***ND1***	3391	G to C (Gly to Ser)	G/S/G/S	G	C			Yes
***ND2***	4491	G to A (Val to Ile)	V/V/I/V	G			A	Yes
	4721	A to G		A		G		Yes
	4769	A to G		A	G		G	Yes
	4820	G to A		G	A			Yes
	4883	C to T		C		T		Yes
	5178	C to A (Leu to Met)	L/T/T/T	C		A		Yes
**tRNA^Cys^**	5802	T to C	T/T/T/T	T		C		Yes
***CO1***	7028	C to T		C	T	T	T	Yes
	7196	C to A		C	A			Yes
***CO2***	7785	T to C (Ile to Thr)	I/I/I/T	T			C	Yes
	8020	G to A		G		A		Yes
**NC_7**	8271-9	9-bp Del	T/S/L/Q	9-bp	9-bp Del			Yes
**tRNA^Lys^**	8343	A to G	A/A/A/A	A	G			Yes
***A6***	8414	C to T (Leu to Phe)	L/F/M/W	C		T		Yes
	8701	A to G (Thr to Ala)	T/S/L/Q	A			G	Yes
	8860	A to G (Thr to Ala)	T/A/A/T	A	G	G	G	Yes
	8964	C to T		C		T		Yes
***CO3***	9300	G to A		G		A		Yes
	9455	A to G		A			G	Yes
	9492	G to C		G	C			Yes
	9540	T to C		T		C	C	Yes
	9824	T to A		T		A		Yes
***ND3***	10398	A to G (Thr to Ala)	T/T/T/A	A		G		Yes
	10400	C to T		C		T		Yes
***ND4***	10873	T to C		T		C	C	Yes
	11719	G to A		G			A	Yes
	11914	G to A		G			A	Yes
***ND5***	12705	C to T		C		T	T	Yes
	13590	G to A		G	A			Yes
***ND6***	14308	T to C		T			C	Yes
	14313	C to T		C		T		Yes
	14587	A to G		A	G			Yes
	14668	C to T		C		T		Yes
***CytB***	14766	C to T (Thr to Ile)	T/S/I/S	C	T	T	T	Yes
	14783	T to C		T		C	C	Yes
	15043	G to A		G		A	A	Yes
	15106	G to A		G	A			Yes
	15301	G to A		G		A	A	Yes
	15326	A to G (Thr to Ala)	T/M/I/I	A	G	G	G	Yes
	15535	C to T		C	T			Yes
**tRNA^Thr^**	15930	G to A	G/G/G/G	G			A	Yes

a Conservation assessment of each mutation/variant is performed through 17 vertebrate mtDNA sequences from NCBI databases including human (H), bovine (B), mouse (M), and *Xenopus laevis* (X), the CI≥75% is regarded as having functional potential

b rCRS: revised Cambridge reference sequence

c Please see Mitomap (www.mitomap.org) database, if the mutation/variant is not reported in Mitomap database, it belongs to ‘novel’.

In fact, the T5802C mutation, as shown in Figure 4, resided at the second base in the anticodon stem, corresponding to conventional position 30 of tRNA^Cys^ [27]. Furthermore, the A8343G mutation occurred at position 54 in the TψC loop of tRNA^Lys^ [28]. In addition, the G15930A mutation disrupted the conserved base-pairing (45G-25C) and may result the failure in mt-tRNA metabolism [29].

### The T5802C, A8343G and G15930A mutations caused the thermodynamic changes of mt-tRNAs

To see whether T5802C, A8343G and G15930A mutations altered the mt-tRNA structure, we performed RNA Fold Webserver programme to predict the MFE structure of mt-tRNAs with and without these mutations (http://rna.tbi.univie.ac.at/cgi-bin/RNAfold.cgi). As can be seen from Table 4, the T5802C, A8343G and G15930A mutations caused a significant thermodynamic alternation of tRNA^Cys^, tRNA^Lys^ and tRNA^Thr^, respectively, suggesting that these mutations may change the secondary structure of mt-tRNA genes, which was critical for the impairment of tRNA functions [30].

**Table 4:** Molecular features of 3 mt-tRNA mutations identified in the present study

tRNA Species	Nucleotide changes	Number of nucleotides in tRNA	Location in tRNA	*G* (wild-type) Kcal/mol	*G* (mutant) Kcal/mol	Disease association
**tRNA^Cys^**	T5802C	30	Anticodon stem	−17.11	−20.38	Deafness; Obesity
**tRNA^Lys^**	A8343G	54	TψC loop	−16.30	−19.38	PD; T2DM; Hypertension
**tRNA^Thr^**	G15930A	45	Anticodon stem	−15.09	−13.50	Deafness; CHD

Abbreviations: CHD, coronary heart disease; PD, Parkinson’s disease; T2DM, Type 2 diabetes mellitus.

### Assessment of the pathogenicity

As shown in Table 5, the total scores of tRNA^Cys^ T5802C, tRNA^Lys^ A8343G and tRNA^Thr^ G15930A mutations were 11, 9 and 9 points, respectively, suggesting that they belonged to ‘definitely pathogenic’ and ‘possibly pathogenic’ at this stage [21].

**Table 5:** Determining the pathogenicity of 3 mt-tRNA mutations identified in the present study

Scoring criteria	T5802C mutation	Score/20	A8343G mutation	Score/20	G15930A mutation	Score/20	Classification
**More than one independent report**	Yes	2	Yes	2	Yes	2	
**Evolutionary conservation of the base-pair**	No changes	2	No changes	2	No changes	2	
**Variant heteroplasmy**	No	0	No	0	No	0	≤6 points: Neutral polymorphisms;
**Segregation of the mutation with disease**	Yes	2	Yes	2	Yes	2	
**Histochemical evidence of mitochondrial disease**	No	0	No	0	No	0	
**Biochemical defect in Complex I, III or IV**	No	0	No	0	No	0	7–10 points: Possibly pathogenic;
**Evidence of mutation segregation with biochemical defect from single-fiber studies**	No	0	No	0	No	0	
**Mutant mt-tRNA steady-state level or evidence of pathogenicity in trans-mitochondrial cybrid studies**	Strong evidence	5	Weak evidence	3	Weak evidence	3	≥11 points: Definitely pathogenic
**Total score**	Definitely pathogenic	11	Possibly pathogenic	9	Possibly pathogenic	9	

### Mutational analysis of *GJB2, GJB3, GJB6* and *TRMU* genes

To examine the roles of nuclear genes (*GJB2, GJB3, GJB6* and *TRMU*) in the phenotypic manifestation of deafness-associated 12S rRNA mutations, we carried out a mutational screening of these genes in affected matrilineal relatives of three pedigrees. However, we failed to detect any variants in *GJB2, GJB3, GJB6* and *TRMU*.

## Discussion

To minimize the incidence of AINSHL, early genetic screening was recommended. Among these detection methods [9–11], the MAS-PCR was a rapid, convenience and inexpensive assay that had been widely used to screen the mtDNA pathogenic mutations. For example, Bi et al*.* had developed a MAS-PCR that can detect Leber’s Hereditary Optic Neuropathy (LHON)-associated 3 primary mutations with high sensitivity [31]. Furthermore, Urata et al*.* performed a quantitative allele-specific PCR for molecular detection of mitochondrial encephalomyopathy with lactic acidosis and stroke-like episodes (MELAS)-associated tRNA^Leu(UUR)^ A3243G mutation [32]. In addition, Scrimshaw et al*.* generated a MAS-PCR to screen the deafness-associated A1555G mutation, which was a simple and reliable method [33]. But their method can detect the A1555G mutation only, without the C1494T mutation. To address this problem, we optimized this MAS-PCR to overcome the shortcomings in these previous reported studies, which had a reasonable high sensitivity and can discriminate the A1555G and C1494T mutations simultaneously [12]. In the present study, by the application of MAS-PCR to screen the 12S rRNA mutations in 500 deaf patients and 300 controls, our results suggested that there were two patients with A1555G mutation (0.4%) and one patient carrying C1494T mutation (0.2%). Further PCR-Sanger sequence confirmed this conclusion (Table 1, *Kappa* = 1.000, *P*<0.01), which had the high sensitivity and specificity.

We further performed clinical, genetic and molecular characterization of three Han Chinese families carrying these 12S rRNA mutations. The hereditary pattern of AmAn hypersensitivity was consistent with maternal transmission, indicating the involvement of mitochondrial dysfunction. As shown in Figure 1, hearing loss was the only clinical phenotype presented in matrilineal relatives but not in other members in these families. The penetrances of hearing loss in HZD501; HZD502 and HZD503 ranged from 22.2% to 42.8% (AmAn included), and 11.1% to 14.3% (AmAn excluded). Compared with previous studies, if the AmAn was included, the penetrances of mitochondrial A1555G-induced hearing loss ranged from 13.0% to 71.4%, with an average of 45.75%. While the AmAn was excluded, the penetrances of A1555G-induced deafness ranged from 8.0% to 51.5%, with an average of 23.85% (Table 6) [34–39]. Whereas in other seven families with C1494T mutation, the penetrances of hearing loss ranged from 6.3% to 42.8% (with AmAn, average: 18.8%). When the effect of AmAn was excluded, the penetrances of deafness ranged from 0 to 14.3%, with the average of 8.3% (Table 6) [40,41]. Moreover, all affected matrilineal relatives in these families exhibited variable severity, age at onset and audiometric configuration of hearing loss, which suggested that the mitochondrial 12S rRNA mutations were not sufficient to produce enough clinical phenotypes, hence, other modified factors including AmAn, nuclear genes, mitochondrial haplogroups or epigenetic modification may contribute to the deafness expression.

**Table 6:** Summary of clinical and molecular data for 15 Chinese families harboring the mitochondrial 12S rRNA mutations

Pedigree number	Number of matrilineal relatives	Penetrance of hearing loss (AmAn included) (%)^a^	Penetrance of hearing loss (AmAn excluded) (%)	mtDNA primary mutation	mtDNA secondary mutation	mtDNA haplogroup^b^	References
**1**	9	22.2	11.1	A1555G	tRNA^Cys^ T5802C	D4b2b	This study
**2**	8	37.5	12.5	A1555G	tRNA^Thr^ G15930A	K1a	This study
**3**	15	66.7	33.3	A1555G	tRNA^Asp^ A7551G	B5a	[34]
**4**	9	66.6	33.3	A1555G	tRNA^Ile^ A4317G	B4c1b2	[35]
**5**	7	71.4	28.6	A1555G	CO2 G7598A	M7b1	[36]
**6**	34	63.6	51.5	A1555G	tRNA^Thr^ T15941C	B4c1c	[37]
**7**	8	25.0	12.5	A1555G	None	D4b2b	[38]
**8**	13	13.0	8.0	A1555G	None	N9a1	[39]
**9**	7	42.8	14.3	C1494T	tRNA^Lys^ A8343G	B4b1c	This study
**10**	39	20.5	12.8	C1494T	tRNA^Tyr^ A5836G	H2b	[40]
**11**	15	6.7	0	C1494T	None	D	[40]
**12**	16	6.3	0	C1494T	None	F1	[40]
**13**	13	15.4	7.7	C1494T	None	D5a2a	[40]
**14**	30	20	13.3	C1494T	None	F1a1	[40]
**15**	10	20	10	C1494T	CO1/tRNA^Ser(UCN)^ G7444A	C4a1	[41]

a Affected matrilineal relatives/total affected matrilineal relatives

b Haplogroup is classified based on the phylotree (http://www.phylotree.org/).

Furthermore, mutations in *GJB2* [42], *GJB3* [43], *GJB6* [44] and *TRMU* [45] were implicated to be associated with hearing impairment. However, the absent of any functional variants in these genes suggested that nuclear modified genes may not play active roles in the clinical expression of deafness-associated 12S rRNA mutations.

Recent experimental studies revealed that mtSNPs or haplogroups may affect the phenotypic manifestation of deafness-associated 12S rRNA mutations [46]. In particular, mtDNA haplogroup B5b specific tRNA^Thr^ G15927A, haplogroup F2 specific *ND5* T12338C, haplogroup B4 specific CO1/tRNA^Ser(UCN)^ G7444A, haplogroup D4 specific tRNA^Arg^ T10454C and tRNA^Ser(AGY)^ C12224T, haplogroup C specific tRNA^Cys^ G5821A, haplogroup Y2 specific tRNA^Glu^ A14693G variants may increase the risk for hearing impairment among the subjects carrying A1555G mutation [47]. Moreover, mtDNA haplogroup F1 specific tRNA^Ala^ T5628C variant was thought to enhance the penetrance and expressivity of C1494T-induced deafness in a large Chinese pedigree [48]. However, Zhu et al*.* analyzed the complete sequences from 13 deaf Chinese families with C1494T mutation and found that their mtDNA belonged to 10 different haplogroups, including haplogroups A, B, D, D4, D4b2, F1, M, M7c, N9a1, H2b, and they believed that mtDNA haplogroup-specific variants may not play an important role in the phenotypic manifestation of the C1494T mutation in those families [40].

In the present study, sequence analysis of the entire mitochondrial genomes of the matrilineal relatives from the three pedigrees (HZD501, HZD502 and HZD503) revealed the presence of A1555G or C1494T mutation, together with sets of mtSNPs belonging to East Asian haplogroup B4b1c, D4b2b and K1a, respectively [18]. Of these, the tRNA^Cys^ T5802C, tRNA^Lys^ A8343G and tRNA^Thr^ G15930A mutations were of special interests. In fact, the T5802C mutation (conventional position 30) disrupted an evolutionary conserved base pairing (30A-40U), converting an A-U to a G-U base pairing on the anticodon stem of tRNA^Cys^ [49]. Nucleotide at position 30 was believed to be important for carrying out effective codon recognition and stability of tRNA [50]. It was interesting to note that the G5540A mutation that also occurred at the same position of tRNA^Trp^ was found to be associated with encephalomyopathy [51]. Furthermore, the A to G mutation at position 8343 affected the first base (conventional position 54) of the TψC loop of tRNA^Lys^. Nucleotide at that position was often chemically modified and thus contributed to the structure and stability of functional tRNA [52]. Importantly, the A14693G mutation, which was also localized at position 54 of tRNA^Glu^, was implicated to modulate the clinical expression of deafness-associated A1555G mutation in a Chinese pedigree [53]. In addition, the G15930A mutation was localized at the anticodon stem of tRNA^Thr^, this mutation disrupted the highly conserved base-pairing (45G-25C) [29]. Moreover, our recent study suggested that the homoplasmic C3275T mutation, which was also located at the same position in tRNA^Leu(UUR)^, was implicated to be associated with LHON [54], polycystic ovary syndrome (PCOS) and metabolic syndrome [55]. In fact, the T5802C mutation was identified in subjects with obesity [56], A8343G mutation appeared in patients with Parkinson’s disease [57], as well as Type 2 diabetes mellitus, atherosclerosis and essential hypertension according to our previous investigation [28], while the G15930A mutation was believed to be associated with coronary heart disease (CHD) [58]. Moreover, bioinformatics analysis revealed that the T5802C, A8343G and G15930A mutations caused the thermodynamic changes of the corresponding tRNAs (Table 4). The pathogenicity scoring system suggested that the T5802C, A8343G and G15930A mutations may be classified as ‘definitely pathogenic’ and ‘possibly pathogenic’ [21]. Thus, the alteration of structure of these tRNAs by the T5802C, A8343G and G15930A mutations may lead to failure in tRNAs metabolism and consequently led to a reduced rate of mitochondrial respiratory chain synthesis [59,60]. Therefore, the mitochondrial dysfunctions, caused by the A1555G or C1494T mutation, may be worsened by T5802C, A8343G and G15930A mutations in these families.

In conclusion, our data indicated that the MAS-PCR was a fast, convenience, cost-effective way for dual-targets identification that can be used in the molecular diagnosis of deafness-associated A1555G or C1494T mutation. Moreover, the tRNA^Cys^ T5802C, tRNA^Lys^ A8343G and tRNA^Thr^ G15930A mutations should be added as risk factors for hearing loss, our study provided novel insight into the molecular pathophysiology of mitochondrial deafness that was manifestated by mitochondrial dysfunction.

## Abbreviations

AINSHL: aminoglycoside-induced and non-syndromic hearing loss
AmAn: aminoglycoside antibiotics
ARMS-PCR: amplification refractory mutation system PCR
CHD: coronary heart disease
CI: conservation index
dB: decibels
DHPLC: denaturing high-performance liquid chromatography
LHON: Leber's Hereditary Optic Neuropathy
MAS-PCR: multiplex allele-specific PCR
MELAS: mitochondrial encephalomyopathy with lactic acidosis and stroke-like episodes
MFE: minimum free energy
mtDNA: mitochondrial DNA
mtSNP: mtDNA single-nucleotide polymorphism
mt-tRNA: mitochondrial tRNA
OXPHOS: oxidative phosphorylation
PCOS: polycystic ovary syndrome
PF: partition function
PTA: pure-tone audiometry
rCRS: revised Cambridge References sequence
